# A descriptive analysis of the Canadian prehospital and transport transfusion (CAN-PATT) network

**DOI:** 10.1016/j.resplu.2022.100357

**Published:** 2023-01-11

**Authors:** Adam Greene, Jan Trojanowski, Andrew W. Shih, Rob Evans, Eddie Chang, Susan Nahirniak, Dallas Pearson, Oksana Prokopchuk-Gauk, Doug Martin, Charles Musuka, Cindy Seidl, Michael Peddle, Yulia Lin, Justin A. Smith, Scott MacDonald, Lindsay Richards, Michael Farrell, Brodie Nolan

**Affiliations:** a AirEvac and Critical Care Operations, British Columbia Emergency Health Services, Vancouver, BC, Canada; b School of Medicine, Cardiff University, Cardiff, Wales, United Kingdom; c Department of Pathology and Laboratory Medicine, Vancouver Coastal Health Authority, Vancouver, BC, Canada; d Centre for Blood Research, University of British Columbia, Vancouver, BC, Canada; e Shock Trauma Air Rescue Service, AB, Canada; f Faculty of Medicine, University of Alberta, Edmonton, AB, Canada; g Alberta Precision Laboratories, Transfusion and Transplantation Medicine, AB, Canada; h Shock Trauma Air Rescue Service, SK, Canada; i Division of Transfusion Medicine, Department of Pathology and Laboratory Medicine, Saskatchewan Health Authority, Saskatoon, SK, Canada; j College of Medicine, University of Saskatchewan, Saskatoon, SK, Canada; k Shock Trauma Air Rescue Service, MB, Canada; l Department of Transfusion Medicine, Shared Health Manitoba, MB, Canada; m Ornge, Mississauga, ON, Canada; n Division of Emergency Medicine, Schulich School of Medicine, Western University, London, ON, Canada; o Precision Diagnostics and Therapeutics Program, Sunnybrook Health Sciences Centre, Toronto, Ontario and Department of Laboratory Medicine and Pathobiology, University of Toronto, Toronto, ON, Canada; p Emergency Health Services LifeFlight, Halifax, Nova Scotia, ON, Canada; q Nova Scotia Health Authority Provincial Blood Coordinating Team, Nova Scotia, Canada; r Department of Emergency Medicine, St. Michael’s Hospital, Unity Health Toronto, Toronto, ON, Canada; s Division of Emergency Medicine, Department of Medicine, University of Toronto, Toronto, ON, Canada

**Keywords:** Prehospital care, Critical care transport, Transfusion

## Abstract

**Objective:**

Out-of-hospital blood transfusion (OHBT) is becoming increasingly common across the prehospital environment, yet there is significant variability in OHBT practices. The Canadian Prehospital and Transport Transfusion (CAN-PATT) network was established to collaborate, standardize, and evaluate the effectiveness of out-of-hospital blood transfusion (OHBT) across Canada. The objectives of this study are to describe the setting and organizational characteristics of CAN-PATT member organizations and to provide a cross-sectional examination of the current OHBT practices of CAN-PATT organizations.

**Methods:**

This was a cross-sectional examination of all six critical care transport organizations that are involved in CAN-PATT network. Surveys were sent to identified leads from each organization. The survey focused on three main areas of interest: 1) critical care transport organizational service and coverage, 2) provider, and crew configurations, and 3) OHBT transfusion practices.

**Results:**

All six surveys were completed and returned. There are a total of 30 critical care transport bases (19 rotor-wing, 20 fixed-wing and 6 land) across Canada and 11 bases have a blood-on-board program. Crew configurations very between organizations as either dual paramedic or paramedic/nurse teams. Median transport times range from 30 to 46 minutes for rotor-wing assets and 64 to 90 minutes for fixed-wing assets. Half of the CAN-PATT organizations started their out-of-hospital blood transfusion programs within the last three years. Most organizations carry at least two units of O-negative, K-negative red blood cells and some organizations also carry group A thawed plasma, fibrinogen concentrate and/or prothrombin complex concentrate. All organizations advocate for early administration of tranexamic acid for injured patients suspected of bleeding. All organizations return un-transfused blood components to their local transfusion medicine laboratory within a predefined timeframe to reduce wastage.

**Conclusions:**

Variations in OHBT practices were identified and we have suggested considerations for standardization of transfusion practices and patient care as it relates to OHBT. This standardization will also enable a robust means of data collection to study and optimize outcomes of patients receiving OHBT. A fulsome description of the participating organizations within CAN-PATT should enhance interpretation of future OHBT studies that will be conducted by this network.

## Introduction

Hemorrhagic shock is one of the leading causes of preventable death in the world, resulting in an estimated 1.9 million deaths per year worldwide.^1^ Trauma is the leading cause of hemorrhagic shock and the foremost cause of death in people under the age of 44, resulting in an estimated 1.5 million deaths per year.^1^, ^2^, ^3^ Other causes of hemorrhagic shock include aneurysmal, gastrointestinal, peri- or post-operative, and obstetrical hemorrhage,^1^, ^4^ resulting in an estimated 400,000 deaths per year.^1^, ^5^, ^6^

The successful resuscitation of any patient in shock requires rapid identification, treatment of the underlying etiology, and restoration of adequate perfusion through interventions centred around optimising the oxygen content of blood to maintain end-organ perfusion. The resuscitation of patients in hemorrhagic shock requires aggressive hemorrhage control to restore the intravascular volume and oxygen-carrying capacity, thereby mitigating the depth and duration of the shock state and repaying the accumulated oxygen debt.^1^

Canada is the second largest country in the world, spanning nearly 10 million sq/km of access- and weather- challenged geography, with most of the population concentrated along the southern border.^7^, ^8^ In Canada, healthcare is largely regionalised, with secondary, tertiary- and quaternary-level care concentrated into regional hubs. While early hemostatic resuscitation is emerging as best practice in select trauma and medical patients, the timely provision of blood components and products during primary (prehospital) and secondary (interfacility) critical care transport (CCT) can significantly impact clinical outcomes. The tyranny of distance disproportionately affects rural, remote, and indigenous communities across Canada. As a result, access to blood products can be limited, and the blood products carried by CCT teams are in some circumstances the first available to critically ill and injured patients.

Despite being widely adopted in military and civilian clinical practice guidelines,^9^, ^10^ the current body of evidence supporting blood transfusion in the prehospital and transport environment is limited and of variable quality.^10^ The randomized control RePHILL trial failed to demonstrate that resuscitation with prehospital red blood cells and lyophilised plasma was superior to 0.9% sodium chloride for trauma related hemorrhagic shock in the civilian population.^11^ The Prehospital Air Medical Plasma (PAMPer) clinical trial showed a nearly 30% reduction in mortality with prehospital plasma transfusion compared to standard care, while the Control of Major Bleeding After Trauma (COMBAT) clinical trial showed no survival improvement.^12^, ^13^ A post-hoc analysis of the COMBAT and PAMPer trials suggested that patients with transport times beyond 20 minutes may benefit from receipt of prehospital plasma, however this study is limited by its observational design.^14^ A recent survey by the European Society of Anaesthesiology found considerable variation in prehospital practice, with little consensus regarding what, how, and when blood products should be deployed in the prehospital and transport environment.^15^ Additionally, there is variation in the types of blood components carried and indications for adjuncts such as calcium administration.^16^

**Fig. 1:**
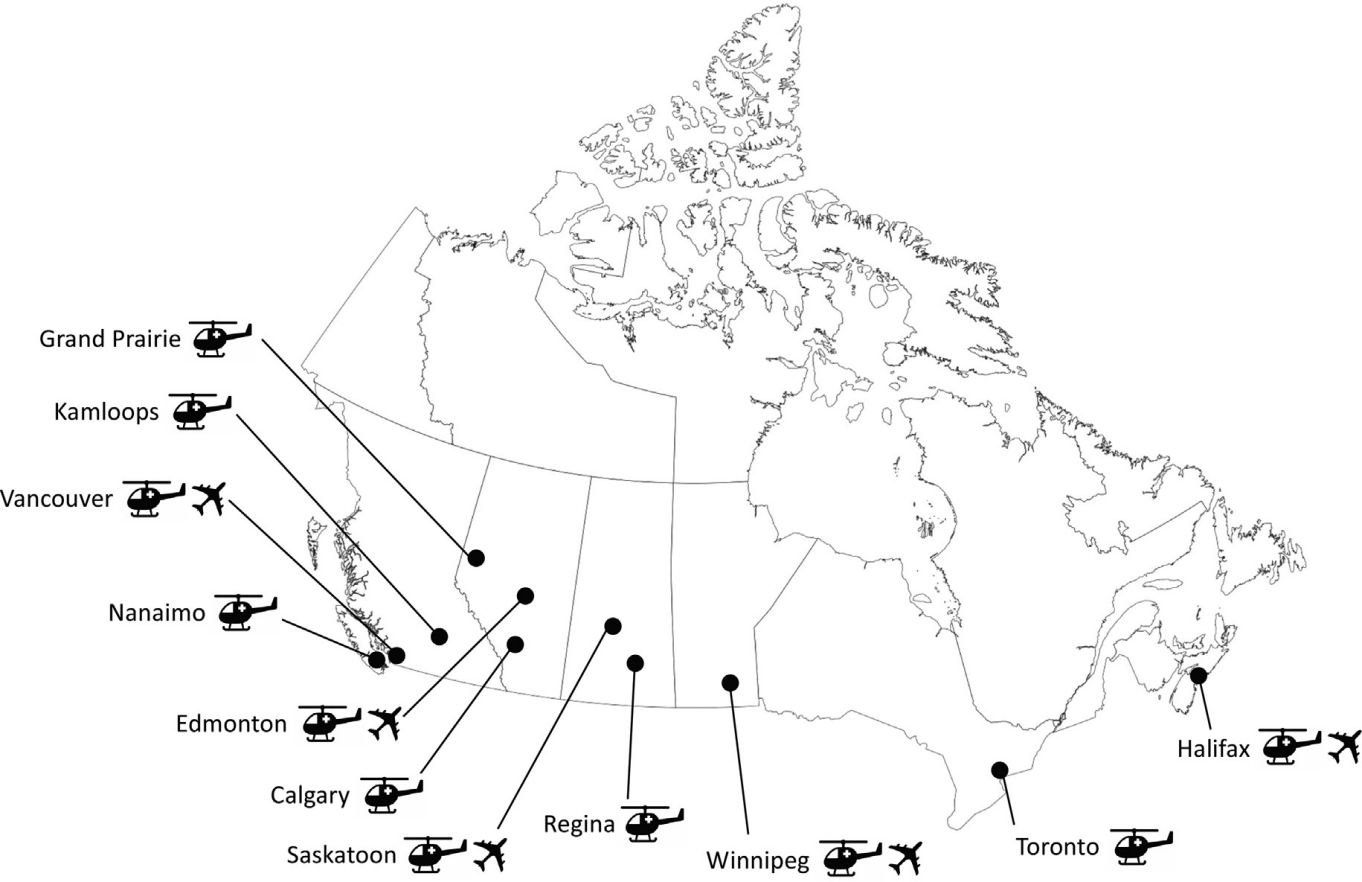
CAN-PATT bases with blood-on-board programs (includes fixed-wing and rotor-wing).

The Canadian Prehospital and Transport Transfusion (CAN-PATT) network was established to collaborate, standardize, and evaluate the effectiveness of out-of-hospital blood transfusion (OHBT) across Canada. Out-of-hospital blood transfusion encompasses two entries: first, the use of blood-on-board critical care transport resources for emergency use, and second, blood issued by a sending hospital for ongoing resuscitation during interfacility transfer. The focus of CAN-PATT is on the former, or the blood-on-board practices of OHBT. In addition, CAN-PATT provides the structure for conducting OHBT research initiatives in a collective, national environment. CAN-PATT consists of six critical care transport organizations across Canada with a total of 30 bases (19 rotor-wing, 20 fixed-wing and 6 land). Some bases have combined use of rotor-wing, fixed-wing and/or land resources). Of these, 11 bases have an active blood-on-board program (Fig. 1). Each organization is responsible for the infrastructure, maintenance, and training for their own OHBT practice; and collects standardized data for quality assurance and to support clinical research activities.

Here, we present data detailing the participating organizations of the CAN-PATT network in preparation for designing and implementing future studies. There are two objectives of this paper. Firstly, to describe the setting and organizational characteristics of CAN-PATT member organizations, thereby addressing potential generalizability and interpretation of future CAN-PATT studies. Secondly, to provide a cross-sectional examination of the current OHBT practices of CAN-PATT organizations.

## Methods

### Study design

This was a cross-sectional examination of the CAN-PATT network. As the information collected was on organizational practices and no personal health information was collected, research ethics board approval was not required.

### Setting

Canada has a publicly funded healthcare system administered at a provincial level. This provincial oversight model is embedded into CCT organizations across Canada, such that CCT provision is unique for each province; however, some interprovincial transport may occur. Critical care transport organizations have adopted slightly different models of governance, with various configurations of providers, dispatch methods, and oversight. Not all Canadian provinces have a dedicated CCT organization, nor do all provinces have established OHBT practices. Critical care transport providers across Canada include physicians of various backgrounds (often emergency medicine, critical care, and anesthesia), registered nurses, and advanced and critical care paramedics.

### Survey development

The survey content was developed by consensus within the group on how best to describe the various CAN-PATT CCT organizations. This process included an initial survey item development based on a literature review and input from transfusion medicine, transport medicine, emergency medicine, and paramedic experts. The survey was initially piloted with a small group of two transport and transfusion medicine experts from two of the CCT organizations involved in CAN-PATT. This resulted in some clarification of survey items, amalgamation of survey items, and introduction of new survey items for the final survey. In the end, the survey focused on three main areas of interest: CCT organizational service and coverage, provider, and crew configurations, and OHBT transfusion practices. The survey was disseminated to each of the identified CCT organizational leads who are also CAN-PATT members. Responses included collaborative discussion between clinical and laboratory team members involved in CCT services within their respective jurisdictions. The final survey had 29 unique items that were filled out electronically via an Excel sheet (Microsoft V16.67). Survey responses were kept confidential until all data collection was complete. Respondents were contacted a second time within 4 weeks if the survey was not completed.

## Results

In total, six surveys were sent out (one to each of the leads from the various CAN-PATT organizations) and all were completed and returned.

### Service coverage and transport characteristics

Each of the CAN-PATT organizations is responsible for critical care transport within their respective provinces. This represents large geographic areas (range: 133,850 to 1,076,395 square kilometers) and service coverage populations (range: 1,180,000 to 14,500,000 people). All organizations have a mix of rotor-wing and fixed-wing assets, with two also having dedicated critical care land assets. The median transport distance ranges from 100 to 183 km for rotor-wing assets and 305 to 544 km for fixed-wing resources (Table 1). Median transport times range from 30 to 46 minutes for rotor-wing assets and 64 to 90 minutes for fixed-wing assets.

**Table 1:** CAN-PATT service and coverage area characteristics (by organization).

**Organization**	**Service Area Population**	**Geography covered (km sq)**	**Annual number of calls (n)**	**Total bases (n)** †	**Rotor-wing bases (n)**	**Fixed-wing bases (n)**	**Critical care land bases (n)**	**Median Transport Distance**	**Median Transport Time**	**All hospitals in area (n)**	**Trauma centres in area (n)**
Ornge (Ontario)	1,45,00,000	10,76,395	20,000	14	7	3	5	**RW:** 101 km	**RW:** 46 min	240	9 adult,
								**FW**: 305 km	**FW**: 64 min **Land**: 45 min		4 pediatric
								**Land**: 35 km			
BCEHS	50,00,000	9,44,000	8,300	7	5	3	0	**FW:** 440 km	**RW:** 29 min	98	3 level I,
								RW: *	FW: *		3 level II
STARS Alberta	60,00,000	5,95,000	1,450	3	3	1	0	**RW**: 125 km	**RW**: 36 min	175	5 level I,
											2 urban trauma centres,
											5 regional level III
STARS Saskatchewan	11,80,000	5,86,710	1,500	3	2	1	0	**RW**: 153 km **FW:** 320 km	**RW**: 43 min	63	2 level I
									**FW**: 90 min		
STARS Manitoba	13,00,000	6,49,950	1,245	2	1	1	0	**RW**: 100 km	**RW**: 30 min	85	1 level I
								**FW**: 544 km	**FW**: 85 min		
EHS LifeFlight (Nova Scotia)	19,23,000	133, 850	1,435	1	1	1	1	**RW**: 187 km	**RW:** 35 min **FW**: 40 min	17	1 level I
								**FW**: 376 km	**Land**: 90 min		

BCEHS: British Columbia Emergency Health Services, STARS: Shock Trauma Air Rescue Service, EHS: Emergency Health Services, RW: rotor-wing, FW: fixed-wing, *: data unavailable. †Some bases share assets and may have both rotor-wing, fixed-wing, and critical care land capabilities.

### Provider configuration

Most organizations within CAN-PATT are staffed with either dual paramedic or a paramedic-nurse configuration with online transport physician oversight (Table 2).

**Table 2:** CAN-PATT provider type (by organization).

**Organization**	**Critical Care Paramedic (n)**	**Advanced Care Paramedic/ALS (n)**	**Primary Care Paramedic/BLS (n)**	**Registered Nurse (n)**	**Physician (n)**	**Typical background of physician**	**Typical Crew Configuration**
Ornge	120	90	10	0	29 adult and 8 pediatric transport physicians	Emergency medicine, critical care	Dual paramedic
BCEHS	55	<5	<5	0	30 adult and 11 pediatric transport physicians	Emergency medicine, critical care	Dual paramedic
STARS Alberta	0	24	0	30	59	Emergency medicine, critical care	Paramedic/Nurse +/- Transport Physician
STARS Saskatchewan	35	0	0	45	32	Emergency medicine, anesthesia, critical care	Paramedic/Nurse +/-Transport Physician
STARS Manitoba	10	0	0	10	17	Emergency medicine, anesthesia, critical care	Paramedic/Nurse +/- Transport Physician
EHS LifeFlight	13	0	0	12	8	Emergency medicine, critical care, trauma	Paramedic/Nurse

BCEHS: British Columbia Emergency Health Services, STARS: Shock Trauma Air Rescue Service, EHS: Emergency Health Services.

### Out-of-hospital blood transfusion programs

Half of the CAN-PATT organizations started their out-of-hospital blood transfusion programs within the last three years (Table 3). The annual number of patients transfused by each of the OHBT programs range from 17 to 102, representing anywhere from <0.5% to 7.0% of total annual patient transports. Ornge and British Columbia Emergency Health Services (BCEHS) do not stock blood and blood products at each base; however, they are planning to expand their OHBT programs to further bases. Most organizations carry at least two units of O-negative, K-negative red blood cells (RBCs). In addition to RBCs, BCEHS carry-two units of group A thawed plasma the Saskatchewan Air Ambulance and Shock Trauma Air Rescue Service (STARS) aircraft carry fibrinogen concentrate and prothrombin complex concentrate (PCC), and STARS Manitoba carries PCC. Currently, freeze-dried or lyophilized plasma and whole blood are not approved for use in Canada.

**Table 3:** CAN-PATT out-of-hospital blood transfusion practices (by organization).

**Organization**	**Start of OHBT program (year)**	**Annual transfusions [from OHBT only] (n, [% total patient calls])**	**Bases with OHBT program n (%)**	**Number of coolers per base**	**Blood products per cooler**	**Blood Group**	**Pharmacologic adjuncts used**	**Inventory exchange frequency**	**Internal cooler temperature monitoring**	**Method of cooler storage**
Ornge	2021	38 patients (<0.5%)	1 (7.1)	2	2 RBCs	O-negative,	TXA,	3 times/week	Yes	Room temperature
						K-negative RBCs	Calcium			
BCEHS	2019	26 patients (<0.5%)	3 (42.9)	2 (Vancouver)	2 RBC + 2 Plasma (Vancouver)	O-negative,	TXA,	2–3 times/week	Yes	Cooler in pharmaceutical-grade fridge
				1 (Nanaimo, Kamloops)	2 RBC (Nanaimo, Kamloops)	K-negative RBCs; Group A Plasma	Calcium			
STARS Alberta	2014	102 patients (7.0%)	3 (100.0)	1	2 RBCs	O-negative,	TXA,	2 times/week	Yes	Room temperature
						K-negative RBCs	Calcium			
STARS Saskatchewan	2013	50 patients (3.3%)	3 (100.0)	2	2 RBCs per cooler + 3rd cooler with Fibrinogen Concentrate and PCC	O-negative,	TXA	3 times/week	Yes	Temperature controlled room
						K-negative RBCs				
STARS Manitoba	2016	59 patients (4.7%)	2 (100.0)	3	2 RBCs per cooler + 3rd cooler with PCC	O-negative,	TXA	3 times/week	Yes	Room temperature
						K-negative RBCs				
EHS LifeFlight	2020	17 patients (1.1%)	1 (100.0)	2	2 RBCs	O-negative,	TXA	3 times/week	Yes	Temperature controlled room
						K-negative RBCs				

BCEHS: British Columbia Emergency Health Services, STARS: Shock Trauma Air Rescue Service, EHS: Emergency Health Services, TXA: tranexamic acid, RBC: red blood cells, PCC: prothrombin complex concentrate.

### Integration with local transfusion medicine laboratory

All organizations return un-transfused blood components to their local transfusion medicine laboratory within a predefined timeframe based on transport cooler validation, to ensure blood component and/or product re-distribution to reduce wastage.

### Training

All organizations provide a combination of in-person and online learning modules regarding safe transfusion practices. These modules and trainings were developed in conjunction with provincial transfusion organizations.

### Indications for OHBT

Indications for initiation of OHBT vary across the CAN-PATT organizations (Table 4). Most require clinical suspicion of significant hemorrhage with physiologic (ie. mean arterial pressure or shock index) or biochemical (ie. hemoglobin, lactate) markers of shock.

**Table 4:** CAN-PATT indications for initiation of blood transfusion (by organization).

**Organization**	**Defined trigger for transfusion**
Ornge	Suspected or confirmed hemorrhage of traumatic etiology AND MAP < 65 or Hb < 70 or with physician order
BCEHS	Suspected or confirmed hemorrhage of traumatic etiology AND a EBTN score > 5; Suspected or confirmed hemorrhage of non-traumatic etiology AND signs of shock OR a Hb < 70 g/L
STARS Alberta	Clinically significant haemorrhage with any of 1) shock index ≥ 1.2, 2) Lactate ≥ 4, 3) Hb < 90
STARS Saskatchewan	Clinically significant haemorrhage with any of 1) shock index ≥ 1.2, 2) Lactate ≥ 4, 3) Hb < 90
STARS Manitoba	Clinically significant haemorrhage with any of 1) shock index ≥ 1.2, 2) Lactate ≥ 4, 3) Hb < 90
EHS LifeFlight	Known or suspected hypovolemic shock related to acute blood loss

BCEHS: British Columbia Emergency Health Services, STARS: Shock Trauma Air Rescue Service, EHS: Emergency Health Services, MAP: mean arterial pressure, Hb: hemoglobin, EBTN: early blood transfusion needs.

## Discussion

This descriptive study has three important findings. First, the optimal configuration of OHBT practices remains undefined. Secondly, there is potential for standardization of OHBT practices across CAN-PATT organizations. Lastly, this study describes the CAN-PATT network as a collaborative for future OHBT research initiatives.

### The optimal configuration of out-of-hospital blood transfusion remains undefined

Every province in Canada that has a provincial critical care transport organization has a sitting member at CAN-PATT. This survey not only highlights the differences in these organizational critical care transport organizations, but also their individual OHBT practices. At the organizational level, variability exists in crew configurations (i.e. dual paramedics vs paramedic/nurse), call volume, and the number and types of assets available. However, one consideration common across all CAN-PATT organizations is that transport distances and times are often quite long. Additionally, all CAN-PATT organizations include and emphasize early administration of tranexamic acid as an adjunct treatment for trauma patients. Although the evidence demonstrating survival benefits to patients receiving OHBT is mixed, injured patients with transport times longer than 20 minutes have a survival benefit when receiving OHBT.^11^, ^12^, ^13^, ^14^ The median transport times for all CAN-PATT organization is above this 20-minute period, suggesting that OHBT is likely to be beneficial for these patients. Additionally, while most of the literature on OHBT is regarding patients in hemorrhagic shock secondary to traumatic sources of hemorrhage, there is also a significant burden of non-traumatic sources of hemorrhage (e.g. gastrointestinal, postpartum hemorrhage) in rural and remote areas of Canada that CCT provides the first opportunity for blood component resuscitation.^17^ This is a relatively unstudied population regarding OHBT practices and future CAN-PATT studies should explore the impact of OHBT on these patients. Of the CAN-PATT organizations only carrying RBCs, many are exploring the use of fibrinogen concentrate, PCC, or whole blood as other OHBT therapies.

### Role for standardization of OHBT practices across CAN-PATT

This study demonstrates significant differences surrounding indications for initiation of OHBT. While many rely on some combination of physiologic and biochemical signs of hemorrhagic shock, we have not identified any standard indication for initiation of OHBT. Developing consistent processes across CAN-PATT organizations could allow for more equal comparisons on OHBT efficacy and safety. Blood supply is harmonized as all blood components and products received by CAN-PATT organizations is sourced from Canadian Blood Services, a national non-profit organization that collects, processes, and distributes all blood donations and blood products for all provinces and territories in Canada (except Québec). Standardization of OHBT across Canada could minimize wastage and protect access to blood components, especially during national blood shortages.

### CAN-PATT as a network for future OHBT research

A recent Delphi study identified high-priority research questions generated from the National Trauma Research Action Plan panel on prehospital and mass casualty trauma care.^18^ Many of these priority research questions surrounded OHBT practices, such as which OHBT strategies reduce mortality, which patients benefit most from OHBT, and what is the efficacy of OHBT in rural and remote populations.^18^ CAN-PATT is well situated to explore these priority research questions at a national level. One of the near-term goals of CAN-PATT is the creation of a national OHBT registry. An OHBT registry would link the out-of-hospital care provided to in-hospital outcomes, providing an essential link to explore many of these priority research questions.

A strength of this survey was that it encompasses the entirety of OHBT organizations within Canada. It provides an oversight of both current national critical care capabilities and current OHBT practices. This study will assist in the planning and implementation of future CAN-PATT trials. Out-of-hospital transfusion practices within each of the CAN-PATT organizations is continuing to evolve as new blood products and components become available. Thus, a limitation of this study is that these practices are likely to change over time. Additionally, this study does not fully articulate some of the more technical transfusion medicine practices, such as specific transport cooler temperature management strategies, validation processes, transfusion administration documentation, or age of red blood cells selected.

## Conclusion

This study presents a cross-sectional examination of the CAN-PATT network and current OHBT practices across Canada. Variations in OHBT practices were identified and we have suggested considerations for standardization of transfusion practices and patient care as it relates to OHBT. This standardization will also enable a robust means of data collection to study and optimize outcomes of patients receiving OHBT. A fulsome description of the participating organizations within CAN-PATT should enhance interpretation of future OHBT studies that will be conducted by this network.

## Financial support

This study was funded by Canadian Blood Services (Grant: 2022 Canadian Blood Services Blood Efficiency Accelerator Program). The funding agency did not have any role in the development, data collection, analysis, or interpretation of the study.

## Author contributions

BN conceived and designed the study and obtained research ethics approval. All authors performed data collection. BN analyzed the data. BN and AG drafted the manuscript, and all authors contributed substantially to its revision. BN takes responsibility for the paper as a whole.

## Funding

This study was funded by Canadian Blood Services (Grant: 2022 Canadian Blood Services Blood Efficiency Accelerator Program). The funding agency did not have any role in the development, data collection, analysis, or interpretation of the study.

## Conflicts of interest

There are no conflicts of interest to declare
